# Comment on Flatscher et al. Impact of Mediterranean Diet on Lipid Composition in the Colaus-PsyColaus Study. *Nutrients* 2023, *15*, 4659

**DOI:** 10.3390/nu16101424

**Published:** 2024-05-09

**Authors:** Patrizia Palumbo, Elena Buzzetti, Ger H. Koek

**Affiliations:** 1 School of Nutrition and Translational Research in Metabolism, Maastricht University, 6229 HX Maastricht, The Netherlands; 2 Division of Metabolic Diseases and Clinical Nutrition, University Hospital of Modena, 41124 Modena, Italy; 3 Department of Medical and Surgical Science for Children and Adults, Division of Medicine and Center for Heredo-Metabolic Diseases of the Liver, University Hospital of Modena “Policlinico”, 41124 Modena, Italy; elena.buzzetti@unimore.it; 4 Department of Gastroenterology and Hepatology, Maastricht University Medical Centre+, 6229 HX Maastricht, The Netherlands; gh.koek@mumc.nl

**Keywords:** Mediterranean diet, food frequency questionnaire, blood lipid levels

With great interest, we read the article by Flatscher et al. that was recently published in Nutrients. In this study, the authors investigated the effect of adherence to the Mediterranean diet (MD) on the serum lipid levels in a population outside the Mediterranean region. The study showed that the MD did not have any notable impact on the serum lipid profile [1].

The investigators focused on two questionnaires evaluating the adherence to the MD: the one developed by Trichopoulou et al. [2] on a Greek population and the version adapted by Vormund et al. [3] to fit the Swiss population, with the assumption of a protective effect of dairy products on cardiovascular or cancer risk. It is important to note that Flatscher et al. employed a prospective design and, with a considerably large study population, did not limit the sample to a healthy population. This distinguishes their study from others [1]. However, the limitations of this study are substantial.

Firstly, as mentioned by the authors, the use of the Food Frequency Questionnaire (FFQ) to measure the participants’ eating habits exclusively at the first followup may have introduced bias regarding the data interpretation. In addition, the questionnaire developed by Vormund et al. for their own purposes had not previously been independently validated. Instead, it was utilized as a general tool to assess adherence to good eating habits [3].

Adhering to the MD is recognized to be a predictor of improved blood lipid levels. This evidence has also been detected in countries that are not in the Mediterranean area, such as the United States [4] or particularly in Northern Europe, where the nutritional preventive claims have been adjusted to align with the Mediterranean pattern using types of foods that are naturally prevalent in the territory (for instance, canola oil instead of olive oil) [5,6].

Moreover, the data related to the evaluation of dietary habits (DHs) were collected in a semi-quantitative way [1]. The most reliable method for assessing DHs is through the collection of a dietary record (DR) [7] for 3 or 7 consecutive days and/or assessing data at different times of the year [8]. Such a method is not devoid of bias, first of all determined by the necessity of including a population of individuals who are literate and highly motivated to participate, as well as the potential simplification of the information or changes in eating habits influenced by the effort involved in maintaining a dietary record [8]. Nonetheless, FFQs provide advantages over other methods, such as low cost and self-administration, and, for that reason, they are extensively used and recognized in clinical research; however, they would be more complete in their quantitative form.

At a glance, it is important to note that this study contradicts the large body of literature that shows how following an MD can lead to a decrease in blood lipid levels and, therefore, to a reduced incidence of cardiovascular disease [5,9,10]. Furthermore, it would be substantial to develop and validate tools that not only provide qualitative information but also quantitative data to enhance the reliability of such tests in Nutrition Research.
